# SARS-CoV-2 Omicron infection, who will be developed into severe/critical diseases?

**DOI:** 10.1186/s12967-023-04207-2

**Published:** 2023-05-20

**Authors:** Mudan Feng, Qing Lin, Jian Xu

**Affiliations:** 1 Department of Infectious Diseases, Affiliated Hospital of North Sichuan Medical College, Nanchong, 637000 China; 2 Department of Infectious Diseases, The people’s hospital of Jiulongpo district, Chongqing, 400050 China; 3 Department of Infectious Diseases, The People’s Hospital of Yubei District of Chongqing City, No. 23, North of Central Park, Yubei District, Chongqing, 401120 China

Dear editor,

Although the coronavirus disease 2019 (COVID-19) pandemic has now entered an endemic phase [1], local epidemics still have an important impact on people’s lives and health. Recently, the COVID-19 epidemic has been on the rise in China. It is extremely meaningful to analyze and summarize the lessons learned from previous waves of the COVID-19 pandemic, which can provide references for nowadays and the future epidemic.

After infection with SARS-CoV-2, the cross-reactivity between virus and host may lead to different clinical outcomes including asymptomatic, mild, moderate, severe, and critical illness [2]. A total of 6228 patients infected with SARS-CoV-2 Omicron were hospitalized between November 12, 2022, and December 11, 2022. Among of them, 95.09% (5922/6228) patients had mild/moderate illness, while 3.03% (189/6228) patients had severe/critical illness. 117 (1.88%) patients with chronic kidney disease (CKD) were in a severe/critical condition due to the aggravation of the disease by SARS-CoV-2 Omicron infection (Fig. 1). The proportion of severe/critical patients was significantly lower than previously reported for SARS-CoV-2 wild-type strains [3].

**Fig. 1:**
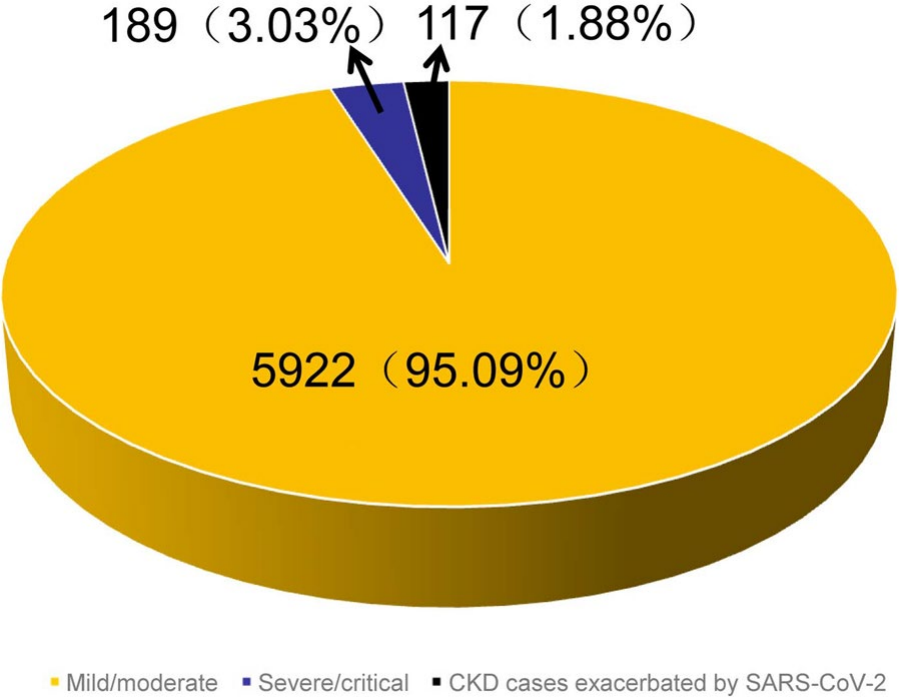
Distribution of disease severity of SARS-CoV-2 Omicron infection

Severe/critical patients consume more medical resources and face greater threats to their lives and safety. Therefore, severe/critical patients are the key group to which attention should be paid in the future. We evaluated the average age of severe/critical patients and found their average age to be significantly higher than that of the mild/moderate group (64.69 ± 17.76 years vs. 48.24 ± 24.05 years, p < 0.01), suggesting that more attention should be given to aged patients, especially those over 65 years old, who are at greater risk of developing severe/critical disease.

We analyzed the pre-existing diseases of 189 severe/critical patients and found that the proportion of severe/critical patients with pre-existing cardiovascular diseases (CD), respiratory diseases (RD), metabolic diseases (MD), gastrointestinal and hepatobiliary diseases (GHD), tumor diseases (TD), urinary diseases (UD), nervous system diseases (ND), infectious diseases (ID), osteoarthritis diseases (OD), and sense organ diseases (SOD) was 50.26%, 46.03%, 46.03%, 26.98%, 23.81%, 23.28%, 20.11%, 15.87%, 4.23%, and 2.65%, respectively, significantly higher than the corresponding proportions of mild/moderate patients (p < 0.01); however, although the proportion of severe/critical patients with pre-existing connective tissue diseases (CTD) and skin diseases (SD) was slightly higher than that of mild/moderate patients, the difference was not statistically significant (p > 0.05) (Fig. 2). The cardiovascular system may be the main target of SARS-CoV-2, and CD (mainly including heart failure, arrhythmia, congenital heart disease, rheumatic heart disease, hypertension, coronary atherosclerotic heart disease, valvular heart disease, cardiomyopathy, and pericardial disease) were the most common diseases in all phases of COVID-19 infection. SARS-CoV-2 infection and the host’s immune response may play key roles in disease progression, and SARS-CoV-2 infection is capable of inducing endothelial inflammation in various organs. A total of 46.03% patients had RD (mainly including pulmonary infection, bronchiectasis, COPD, bronchial asthma, pulmonary hypertension, interstitial lung disease, and respiratory failure) and MD (mainly diabetes, thyroid dysfunction, metabolic syndrome, osteoporosis, and electrolyte disorders). This may be because the body is already in an inflammatory state in these patients. Inflammatory biomarkers including IL-6, C-reactive protein, D-dimer, ferritin, and procalcitonin are elevated in patients with severe/critical SARS-CoV-2 infection [4]. Therefore, we have put forth the concept of “bi-anti” for the early stage of SARS-CoV-2 infection [5], which is of practical significance for patients with the above mentioned underlying diseases. “Bi-anti” therapy may prevent a large number of patients from being hospitalized and thus save limited medical resources. Our findings suggest that a detailed understanding of a SARS-CoV-2 patient’s pre-existing diseases is critical for determining the likelihood of a patient progressing to severe/critical disease.

**Fig. 2:**
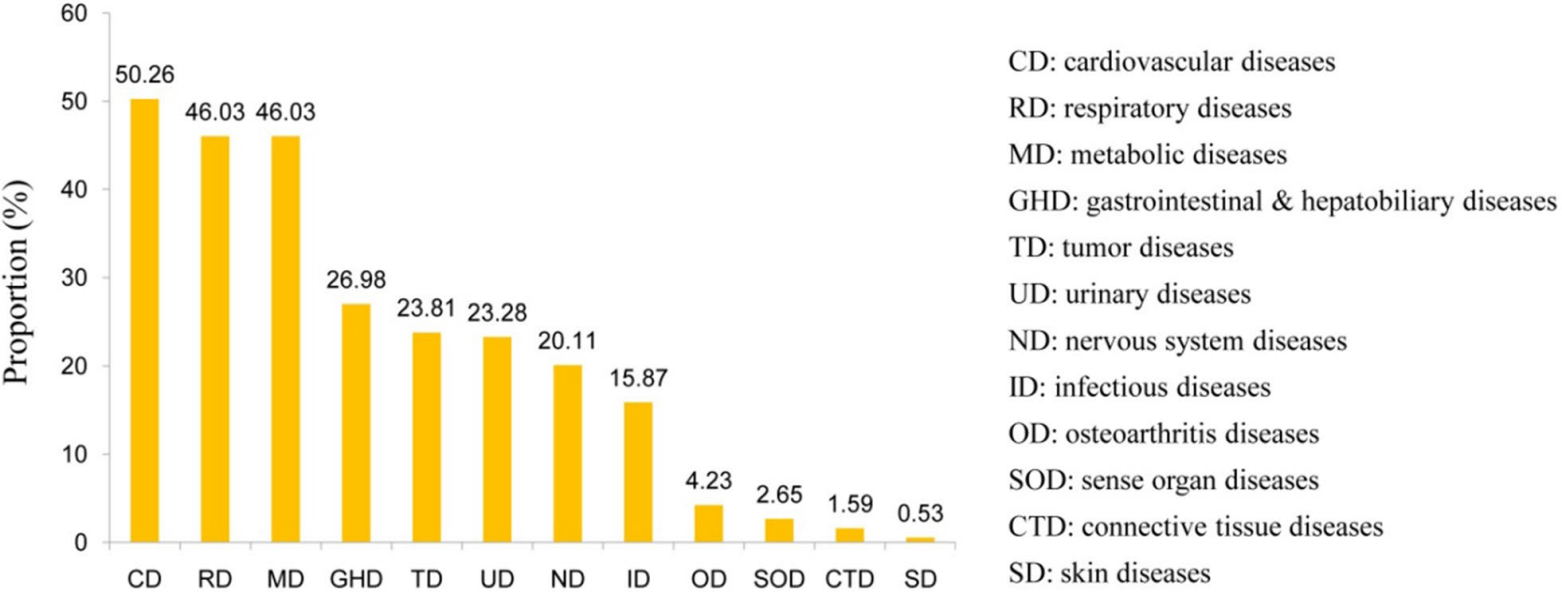
Pre-existing comorbidities in severe/critical patients with SARS-CoV-2 Omicron infection

Overall, COVID-19 is assumed to be a systemic disease, with more than one organ involved in disease progression. Pre-existing diseases can further promote the progression of SARS-CoV-2 infection and lead to severe/critical outcomes. Patients with pre-existing CD, RD, and MD comprise the main population that develops severe/critical disease after SARS-CoV-2 Omicron infection. Therefore, it is necessary to pay special attention to disease prevention and control in these high-risk populations.

## Data Availability

The original data in this study can be obtained from the corresponding author upon reasonable request.
